# Long non-coding RNA NORAD promotes pancreatic cancer stem cell proliferation and self-renewal by blocking microRNA-202-5p-mediated ANP32E inhibition

**DOI:** 10.1186/s12967-021-03052-5

**Published:** 2021-09-22

**Authors:** Yu-Shui Ma, Xiao-Li Yang, Yu-Shan Liu, Hua Ding, Jian-Jun Wu, Yi Shi, Cheng-You Jia, Gai-Xia Lu, Dan-Dan Zhang, Hui-Min Wang, Pei-Yao Wang, Fei Yu, Zhong-Wei Lv, Gao-Ren Wang, Ji-Bin Liu, Da Fu

**Affiliations:** 1 Department of Nuclear Medicine, Shanghai Tenth People’s Hospital, Tongji University School of Medicine, No. 301, Yanchang Middle Road, Jing’an District, Shanghai, 200072 China; 2 Cancer Institute, Affiliated Tumor Hospital of Nantong University, Nantong, 226631 China; 3 Department of Pathology, Affiliated Tumor Hospital of Nantong University, Nantong, 226631 China; 4 Department of Radiotherapy, Affiliated Tumor Hospital of Nantong University, Nantong, 226631 China; 5 Nantong Haimen Yuelai Health Centre, Haimen, 226100 China

**Keywords:** Pancreatic cancer, Pancreatic cancer stem cells, Long non-coding RNA NORAD, microRNA-202-5p, ANP32E, Self-renewal, Proliferation

## Abstract

**Background:**

Cancer stem cells (CSCs) are key regulators in the processes of tumor initiation, progression, and recurrence. The mechanism that maintains their stemness remains enigmatic, although the role of several long noncoding RNAs (lncRNAs) has been highlighted in the pancreatic cancer stem cells (PCSCs). In this study, we first established that PCSCs overexpressing lncRNA NORAD, and then investigated the effects of NORAD on the maintenance of PCSC stemness.

**Methods:**

Expression of lncRNA NORAD, miR-202-5p and ANP32E in PC tissues and cell lines was quantified after RNA isolation. Dual-luciferase reporter assay, RNA pull-down and RIP assays were performed to verify the interactions among NORAD, miR-202-5p and ANP32E. We then carried out gain- and loss-of function of miR-202-5p, ANP32E and NORAD in PANC-1 cell line, followed by measurement of the aldehyde dehydrogenase activity, cell viability, apoptosis, cell cycle distribution, colony formation, self-renewal ability and tumorigenicity of PC cells.

**Results:**

LncRNA NORAD and ANP32E were upregulated in PC tissues and cells, whereas the miR-202-5p level was down-regulated. LncRNA NORAD competitively bound to miR-202-5p, and promoted the expression of the miR-202-5p target gene ANP32E thereby promoting PC cell viability, proliferation, and self-renewal ability in vitro*,* as well as facilitating tumorigenesis of PCSCs in vivo.

**Conclusion:**

Overall, lncRNA NORAD upregulates ANP32E expression by competitively binding to miR-202-5, which accelerates the proliferation and self-renewal of PCSCs.

**Supplementary Information:**

The online version contains supplementary material available at 10.1186/s12967-021-03052-5.

## Background

Pancreatic cancer (PC), a highly fatal disease causing over 200,000 deaths worldwide every year [1]. This high morbidity is due to the tumor’s aggressiveness and the lack of markers or symptoms enabling timely diagnosis and treatment. Therefore, the majority of PC patients are diagnosed at a late stage, when tumors have already metastasized towards distant organs [2, 3]. The main causes of the high mortality of PC are cancer resistance to existing therapies, as well as the occurrence of metastasis that precedes diagnosis [4]. In this regard, it is important is to probe the mechanism of PC progression, if we are to develop more effective early diagnosis and treatment methods [5].

As previously demonstrated, the lncRNAs are engaged in a wide range of processes, including proliferation, migration and apoptosis [6, 7]. Besides, the mechanism whereby lncRNAs participate in cancer growth, cancer stem cells (CSCs), and chemoresistance in PC has recently been illustrated [8]. More specifically, the upregulation of Cdc2 by lncRNA SPRY4-IT1 promoted cell proliferative and invasive capabilities in PC [9], and conversely, the knockdown of lncRNA MIR115HG regulated miR-802 expression to inhibit PC cell viability, and promote cell cycle arrest, and apoptosis [10]. Recently, it has emerged that NORAD can promote the expression of SIP1, thereby inducing the promotion of cell proliferative and invasive abilities in cervical cancer [11]. Moreover, NORAD boosts colorectal cancer cell proliferation, migration, and invasion by means of inhibiting microRNA-202-5p (miR-202-5p) expression [12]. However, much remains to be learned about the effects and biological mechanisms of NORAD in PC According to recent research, miR-202-5p acts as a tumor-suppressor in the context of breast cancer [13] and colorectal carcinoma [14]. Besides, enforced expression of miR-202 is capable of significantly reducing the epithelial-to-mesenchymal phenotypic characteristics of parenchymal PC cells [15]. According to the bioinformatics website, miR-202-5p emerged a downstream miRNA for NORAD, while itself potentially targeting ANP32E. Consequently, we designed our investigation with the purpose of verifying the role of the NORAD/miR-202-5p/ANP32E axis in regulating the biology of PC stem cells (PCSCs).

## Methods

### Ethics statement

The Ethics Committee of Shanghai Tenth People’s Hospital, Tongji University School of Medicine ratified our study. Written informed consents were acquired from patients before their participation in this study. All experimental methods abided by the *Declaration of Helsinki*. All animal studies were undertaken in accordance with the recommendations in the Guide for the Care and Use of Laboratory Animals issued by US National Institutes of Health.

### Clinical sample collection

Cancer and adjacent normal tissues (more than 2 cm away from tumor margins) were surgically acquired from 28 patients (18 males, 10 females; at the age of 39–72 years with a mean age of 54 years) who were pathologically confirmed PC at Shanghai Tenth People's Hospital, Tongji University School of Medicine from May 2016 to December 2017. The patients enrolled had not received either local or systemic treatment prior to the operation. There were nine cases at the stage I, five cases at the stage II and 14 cases at the stage III. The specimens were assessed histopathologically by the hospital pathology department with detailed clinical data were recorded. All specimens were frozen in liquid nitrogen in a quick manner and stored at controlled temperature of − 80 °C for later analysis.

### Cell culture

One normal human pancreatic ductal epithelial cell line HPDE6-C7 (HZ-H296; Shanghai Huzhen Biotechnology Co., Ltd., Shanghai, China) and three PC cell lines BxPC-3, MIAPaCa-2, and PANC-1 (ATCC, Manassas, VA, USA; www.atcc.org) were used. These cells were subjected to culture with the RPMI 1640 medium (consisted of 10% FBS, 100 U/mL streptomycin and 100 U/mL penicillin) at controlled temperature of 37 °C under 5% CO_2_, with the medium being renewed every 2 days. Upon growing to 80–90% confluence, cells were passaged, and exponentially growing cells were used for subsequent experiments. The expression of NORAD in cell lines was tested by RT-qPCR; the PANC-1 cells had the highest NORAD expression and were consequently selected for further study.

### Cell treatment

Sequences for NORAD, miR-202-5p and ANP32E were obtained from the NCBI, and Shanghai Sangon Biological Engineering Technology & Services Co., Ltd. (Shanghai, China) was entrusted with the construction of plasmids including miR-202-5p mimic, small interfering RNA (si)-ANP32E, ANP32E, si-NORAD, NORAD, and corresponding negative controls (NCs) by using pCMV-Flag-N/C plasmid vector.

The third generation of cells were trypsinized and seeded in plates with 24 wells to form monolayer cells. The cells were divided into two parts and subjected to transfection using Lipofectamine 2000 reagent. One portion of the cells was transduced using miR-202-5p mimic, si-ANP32E and ANP32E, alone or in combination. The other portion of cells was treated with si-NORAD, NORAD, miR-202-5p mimic, either alone or in combination. Finally, after 48-h transfection, to screen stably-transfected cells, cells were maintained for 4 weeks under standard condition in G418 (1000–2000 μg/mL) medium, which was renewed every 3–5 days.

### Bioinformatics prediction and dual-luciferase reporter assay

WT luciferase reporter plasmid ANP32E (ANP32E-WT-Luc) containing WT ANP32E sequence and the MUT luciferase reporter plasmid ANP32E (ANP32E-Mut-Luc) containing MUT ANP32E sequence were all from Shanghai Genechem Co., Ltd. (Shanghai, China). Thereafter, 293T cells underwent cotransfection with miR-202-5p mimic or miR-202-5p mimic-NC and reporter vectors with the use of Lipofectamine™ 2000. After incubation for 24 h, luciferase activity was assessed at 560 nm by Dual-Luciferase Reporter Assay Kit and a microplate reader (Thermo MK3).

WT and MUT primers of NORAD were designed and synthesized by Sangon. Total RNA content of PANC-1 cells was extracted and amplified by means of PCR with WT and MUT primers. Hind III and Bgl II enzyme endonuclease sites were added at both ends of the amplified products. pGL3-Basic luciferase reporter vector (Promega) was then digested by restriction endonuclease Hind III/Bgl II, and the large fragments were recovered by electrophoresis. Ligase 4 was linked the amplified target gene and vector to obtain NORAD-WT-Luc and NORAD-MUT-Luc plasmids, which were subsequently transformed into the *E. coli* competence sequence. After colony identification by PCR, the plasmids were extracted from the colony shaker kit containing the target fragment and sequenced. Other procedures were the same as described above.

### RNA-pull down assay

Cells were subjected to transfection with the use of 50 nM biotin-labeled Bio-miR-202-5p-WT and Bio-miR-202-5p-MUT. The cell lysates were incubated with RNase-free BSA and yeast tRNA precoated Dynabeads Streptavidin Magnetic Beads. The enrichment of NORAD was measured by RT-qPCR.

### RIP assay

PANC-1 cells were initially lysed in RIP lysis solution and centrifuged at 14,000 rpm at 4 °C for 10 min. The supernatant was harvested, a portion of which was removed as input while the other was probed with antibodies against rabbit anti-human Ago2 (ab186733, 1: 50, Abcam) and rabbit anti-human IgG (ab109489, 1: 100, Abcam, taken as NC), with SNRNP70 (Millipore) used as a positive control, for co-precipitation. At last, the immunoprecipitated RNA was isolated and analyzed by means of RT-qPCR.

### Fluorescence in situ hybridization (FISH)

Cells post 24-h transfection in each group were detached with trypsin by shaking for 5 min, and then centrifuged for 2–3 min in 1.5 mL Eppendorf (EP) tubes. The cells were fully mixed with the pre-cooled CER I and lysed on ice for 10 min. Additional spinning was conducted with precooled CER II, followed by incubation and centrifugation. Next, the supernatant containing cytoplasmic components was transferred into a new EP tube and then stored at − 80 °C for later analysis. Afterwards, the supernatant was spun with pre-cooled nuclear extraction reagents and incubated. Then, it was remixed for 15 s at intervals of 10 min and finally centrifuged for 10 min at 4 °C. Finally, the supernatant containing the nuclear fraction was put into fresh EP tubes, and stored at − 80 °C.

The coverslips were dried and fixed, after which cells were subsequently treated with protease K, DEPC-4% paraformaldehyde, then acetic acid, and incubated with 200 μL pre-hybridization solution for 1 h at room temperature. After that treatment, 250 μL hybridization solution containing 0.1–0.2 ng/μL probe was added for a further incubation at 65 °C for 14 h. The cells were washed, sealed and finally incubated with anti-DIG-AP Fab antibody (diluted 1:5000 din Buffer B2) overnight at 4 °C. Thereafter, the coverslips were developed with freshly prepared BCIP/NBT solution for 3–24 h in the dark.

### RT-qPCR

Total RNA content of PC cells was extracted by TRIzol reagent, and the purity and concentration of the extracted RNA were determined by NanoDrop ND-1000. Thereafter, cDNA was synthesized with a PrimeScript RT reagent Kit, while RNA was converted to cDNA by a One Step PrimeScript MicroRNA Gene Synthesis Kit. RT-qPCR of the product was implemented using a Quanti-Tect SYBR Green PCR kit on the ABI7500 quantitative PCR system. With U6 and GAPDH serving as the internal reference, relative expression pattern of each target gene was measured by means of 2^−ΔΔCt^ method. PCR primer sequences are listed in Additional file 1: Table S1.

### Western blot analysis

After 72 h of transfection, total protein was extracted and the concentration was assessed by a bicinchoninic acid kit. All the protein lysates were separated using 10% SDS-PAGE, transferred onto a polyvinylidene fluoride membrane, and sealed by 5% skimmed milk powder. After that, the membrane underwent overnight probing at 4 °C with primary antibodies, namely rabbit anti-human antibodies to cleaved-caspase 3 (1:1000, #9665, Cell Signaling Technology, Beverly, MA, USA), cleaved-caspase 9 (1:1000, #9508, Cell Signaling Technology), PARP1 (1:1000, ab32064, Abcam), Oct4 (1:1000, ab181557, Abcam), Nanog (1:200, ab21624, Abcam), and Sox2 (1:1000, #14962, Cell Signaling Technology). Thereafter, the membrane underwent re-probing with HRP-conjugated secondary goat anti-rabbit for 1 h at 37 °C. Finally, the membrane was visualized with enhanced chemiluminescence reagent (Pierce). Ratio of the gray value of target bands to that of the internal reference GAPDH (1:2500, ab9485, Abcam) band represents the relative protein expression.

### Aldefluor assay

Cells post 24-h transfection were resuspended in Aldefluor buffer to adjust the density to 1 × 10^6^ cells/mL. The activity of aldehyde dehydrogenase (ALDH), a stem cell marker, was detected by an Aldefluor kit according to the instructions. The cells were subjected to incubation at 37 °C for 25 min with 15 μM ALDH specific inhibitor exogenous 4-(diethylamino)benzaldehyde (DEAB) and 0.15 μM ALDH substrate. Then the activity of ALDH was measured by a flow cytometer.

### MTT assay

Exponentially growing cells were cultured with 20 μL of MTT (5 mg/mL) at 0, 12, 24, 48 and 72 h after transfection for 4 h in the dark. Cells of each well were supplemented with 150 μL dimethylsulfoxide and placed on a shaking table for 10 min, and the OD value was then measured by means of a microplate reader (DG5031, Shanghai Kehuai Instruments Co., Ltd., Shanghai, China) at 490 nm.

### Flow cytometry

After 24 h of transfection, cells were detached with trypsin without EDTA and centrifuged. After that, collected cells were fixed by addition of 3 mL pre-cooled 70% ethanol, centrifuged, and stained with 0.5 mg/mL propidium iodide (PI) staining solution, followed by detection by a flow cytometer at more than 575 nm. Apoptosis rate of PC cells was assessed by an Annexin V-FITC/PI double staining kit (556547, Shanghai Solja Technology Co., Ltd., China). After centrifugation, cells were resuspended in pre-cooled 1× phosphate buffer saline, centrifuged at 200 rpm for 5–10 min and resuspended in 300 µL 1× binding buffer. Next, the cells were incubated with 5 µL of Annexin V-FITC and stained with 5 µL PI, followed by analysis with a flow cytometer (Cube6, Sysmex Partec, Am Flugplatz, Görlitz, Germany). FITC was detected at 480 and 530 nm, while PI at a wavelength greater than 575 nm. The proportion of stem cell markers CD24+ and CD44+ cells was then calculated. Cells were incubated with FITC-conjugated CD44 (mouse anti-human, BD Biosciences, 555478), and phycoerythrin (PE)-conjugated CD24 (mouse anti-human, BD Biosciences, 555428), along with their corresponding isotype controls (BD Biosciences, 555742 and 55554) for cell surface staining, washed twice with the use of PBS, and resuspended in PBS for analysis/sorting.

### Colony formation assay

PC cells post 24-h transfection were detached with 0.25% trypsin and triturated into single cell suspension. This single-cell suspension was plated in plates with 6 wells (1 × 10^4^ cells/mL) and grown for 2 weeks under standard condition. When cell colonies were observed by naked eye, the culture was halted and the cells underwent 3.7% methanol fixation for 10 min and 0.1% crystal violet staining for 10–30 min. After staining and washing, the cells were photographed and the number of clones (> 50 cells) per well was counted using the Image J software for statistical analysis [16].

### Sphere formation assay

Cells post 24-h transfection were plated in ultralow attachment plates with 24 wells at a density of 1000 cells/well in serum-free DMEM/F-12 medium containing B27 (1:50), 20 ng/mL basic fibroblast growth factor, and 20 ng/mL epidermal growth factor. The number of microspheres formed within 7 days was counted, and the colony formation ratio was calculated based on a factor of 1000.

### Xenograft tumors in nude mice

BALB/c mice aged 5 weeks (equal numbers of male and female) were randomly grouped into 13 groups (12 for each group). The mice were housed under room temperature conditions at a stable humidity of 50–60% under a 12-h light/dark cycle with free access to drinking water. For tumor propagation analysis, 1.5 × 10^6^ cells resuspended in 0.1 mL serum-free DMEM was mixed with 0.1 mL Matrigel and injected subcutaneously into the back of nude mice. After 3 days, a second cell suspension of the same volume was injected at the same site. Tumor formation and volume were observed every 2 days after injection. Four weeks later, mice were euthanized, thus at 5 weeks after tumor innoculation. The weight and volume of the tumors were measured. The volume was then calculated as the calculation (length × width^2^)/2.

### Statistical analysis

The measurement data described as mean ± standard deviation and SPSS 21.0 software was used to analyze the data. The statistical significance was measured using paired *t*-test, unpaired *t*-test, one-way ANOVA with Tukey’s multiple comparisons test and two-way ANOVA or repeated measures ANOVA, followed by Bonferroni post hoc test for multiple comparisons. A value of *p* < 0.05 denotes statistical significance.

## Results

### Bioinformatics analysis predicts that NORAD competitively binds to miR-202-5p to increase ANP32E expression, thus indicating an involvement in PC development

**Fig. 1:**
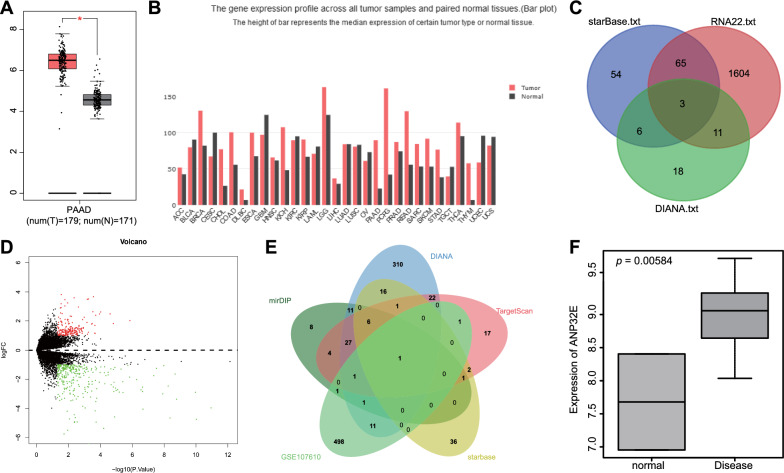
LncRNA, miRNA and mRNA expression profiles in PC. **A** Expression of NORAD in PC where the X axis represents the grouping and the Y axis represents the NORAD expression; **B** Expression of NORAD in all patient tumor samples and paired normal tissues (black represents normal tissues, and red represents tumor samples); **C** Prediction of downstream miRNAs of NORAD (three circles in the figure represent the prediction results of the three databases, respectively, and the middle part represents their intersection); **D** Volcano plot of expression of differentially expressed genes in PC-related datasets, where the X axis denotes differential log10 *p* value and the Y axis denotes log FoldChange. Each point in the plot represents a gene, where red dots represent upregulated genes while green dots represent down-regulated genes; **E** Prediction of target genes of miR-202-5p (the three circles in the figure represent the prediction results of the three databases respectively, and the middle part represents their intersection); **F** Expression of ANP32E in GSE107610 (the X axis represents the tumor samples and normal tissues and the Y axis represents the level of ANP32E)

In an attempt to identify eligible lncRNAs in PC, we analyzed data in microarray expression profiles. According to the GEPIA database (http://gepia.cancer-pku.cn/), NORAD was found to have expression in PC (Fig. 1A), and in most other cancers (Fig. 1B). Following Venn diagram analysis of the downstream miRNAs of NORAD predicted by the starBase (http://StarBase.sysu.edu.cn/index.php), RNA22 (https://cm.jefferson.edu/rna22/) and DIANA (http://carolina.imis.athena-innovation.gr/diana_tools/web/index.php?r=lncbasev2/index) databases, three miRNAs were found at the intersection, including miR-202-5p, miR-496, and miR-485-3p (Fig. 1C). Existing literature has shown that miR-202-5p is involved in the occurrence and development of PC [15], and that NORAD overexpression can inhibit the expression pattern of miR-202-5p [17]. Therefore, miR-202-5p was selected as the target gene for follow-up research. Additionally, the downstream targets of miR-202-5p were predicted by means of mirDIP, DIANA, TargetScan and starBase databases. At the same time, through the PC-relevant dataset GSE107610 of the GEO database (https://www.ncbi.nlm.nih.gov/geo/), 514 differentially expressed genes were obtained (Fig. 1D). Intersection analysis on the predicted results of the target genes of miR-202-5p and differentially expressed genes yielded ANP32E (Fig. 1E), which was highly expressed in the GSE107610 dataset (Fig. 1F). The aforementioned results indicate NORAD may competitively inhibit miR-202-5p expression and consequently promote the expression of ANP32E, thus participating in PC.

### Upregulated NORAD and ANP32E and downregulated miR-202-5p are determined in PC tissues and cells

**Fig. 2:**
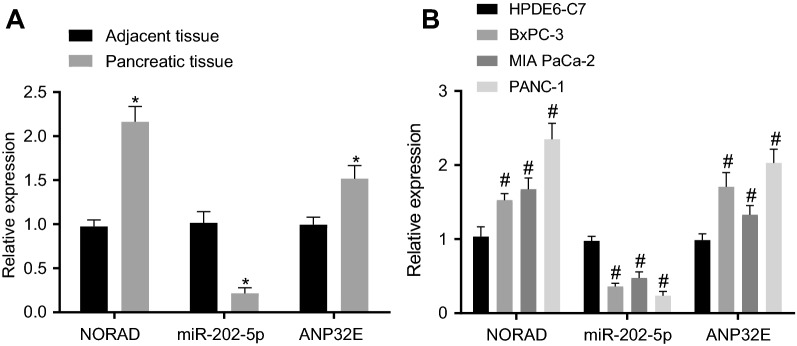
NORAD and ANP32E are highly expressed and miR-202-5p is poorly expressed in PC tissues and cells. **A** Expression of NORAD, ANP32E and miR-202-5p in PC tissues and adjacent normal tissues (n = 28); **B** Expression of NORAD, ANP32E and miR-202-5p in HPDE6-C7, BxPC-3, MIAPaCa-2, and PANC-1 cell lines; * *p* < 0.05 vs*.* the adjacent normal tissues; # *p* < 0.05 vs. HPDE6-C7 cell line; Measurement data were expressed as mean ± standard derivation; data between PC tissues and adjacent normal tissues were compared by paired *t*-test, and measurement data among multiple groups were analyzed by one-way analysis of variance with Tukey’s post hoc test. The experiment was repeated three times

To investigate the role of NORAD, miR-202-5p, and ANP32E in PC, we measured their expression in PC samples and cell lines. In relation to adjacent normal tissues, PC tissues exhibited higher expression of NORAD and ANP32E and lower miR-202-5p expression (Fig. 2A). In addition, NORAD and ANP32E exhibited significantly high expression in the three PC cell lines (BxPC-3, MIAPaCa-2, and PANC-1) relative to HPDE6-C7 cell line, but the expression of miR-202-5p was reduced. Among the three PC cell lines, PANC-1 showed the highest expression of NORAD (Fig. 2B), and was thus selected for subsequent experiments. Furthermore, PANC-1 cells with CD24^+^CD44^+^ESA^+^ were selected as PCSCs (80%).

### miR-202-5p suppresses the viability, proliferation and stemness of PCSCs by inhibiting ANP32E expression

**Fig. 3:**
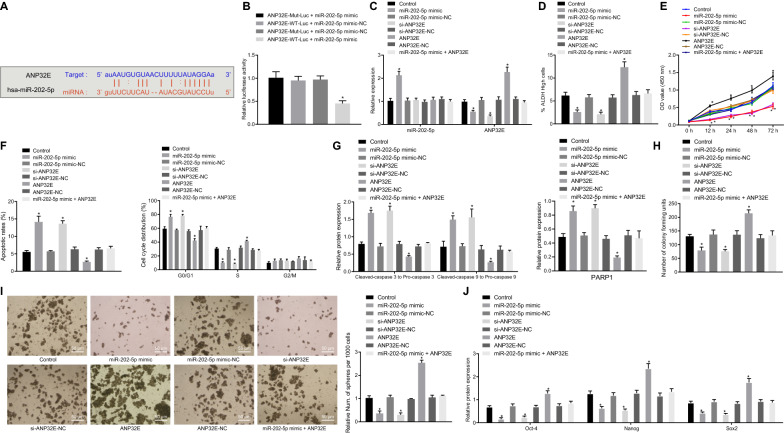
miR-202-5p inhibits the viability, proliferation, and self-renewal of PCSCs, and promotes cell apoptosis by suppressing ANP32E expression. **A** Potential target gene of miR-202-5p predicted by mirDIP, DIANA, TargetScan and starBase databases; **B** Targeting relationship between miR-202-5p and ANP32E measured by dual-luciferase reporter assay (* *p* < 0.05 vs. cells co-transfected with ANP32E-WT-Luc and miR-202-5p mimic-NC, ANP32E-MUT-Luc and miR-202-5p mimic, ANP32E-MUT-Luc and miR-202-5p mimic-NC). PCSCs were treated with ANP32E, si-ANP32E and/or miR-202-5p mimic. **C** miR-202-5p expression and the mRNA expression of ANP32E in PCSCs measured by RT-qPCR; **D** ALDH activity of PCSCs assessed by Aldefluor assay, where mock means a NC with the addition of DEAB (a specific inhibitor of ALDH enzyme); **E** Proliferation of PCSCs detected by MTT; **F** Apoptosis and cell cycle changes of PCSCs measured by flow cytometry; **G** Protein expression of PARP1 and the ratios of cleaved-caspase3 to pro-caspase3 and cleaved-caspase9 to pro-caspase9 in PCSCs detected by Western blot analysis; **H** Colony formation of PCSCs assessed by colony formation assay; **I** Self-renewal ability of PCSCs detected by sphere formation assay (200×); **J** Protein expression of Oct4, Nanog, Sox2 in PCSCs measured by Western blot analysis; * *p* < 0.05 *vs.* cells without treatment. Measurement data were expressed as mean ± standard derivation. Data among multiple groups were analyzed by one-way analysis of variance with Tukey’s post hoc test, and data comparison among multiple groups at different time points was conducted using two-way analysis of variance with Bonferroni post hoc test. The experiment was repeated three times

The mirDIP available at http://ophid.utoronto.ca/mirDIP/index.jsp#r?tdsourcetag=s_pctim_aiomsg, DIANA available at http://diana.imis.athena-innovation.gr/DianaTools/index.php?r=microT_CDS/index&tdsourcetag=s_pctim_aiomsg, TargetScan available at http://www.targetscan.org/vert_71/?tdsourcetag=s_pctim_aiomsg and starBase available at http://StarBase.sysu.edu.cn/index.php databases predicted ANP32E as a potential target gene of miR-202-5p (Fig. 3A). Meantime, the luciferase activity of cells co-transfected with ANP32E-WT-Luc and miR-202-5p mimic was remarkably inhibited, while no alteration occurred in that of cells co-transfected with ANP32E-MUT-Luc and miR-202-5p mimic (Fig. 3B).

Transfection efficiency of cells with miR-202-5p mimic and si-ANP32E was confirmed by RT-qPCR, the results of which illustrated a decreasing trend in the cellular ANP32E expression upon miR-202-5p mimic treatment, while the expression of miR-202-5p was dramatically increased; the expression of ANP32E in the cells transfected with si-ANP32E was remarkably reduced, but was elevated in the cells treated with ANP32E (Fig. 3C).

Based on the effects of DEAB treatment, the ALDH-positive cell population was divided (Additional file 2: Figure S1A, B), and then analyzed by Aldefluor, MTT assay, flow cytometry, colony formation assay, sphere formation assay, and Western blot analysis. Relative to the cells without treatment, the cells with miR-202-5p mimic or si-ANP32E exhibited a decreased proportion of ALDH high-activity cells, inhibited proliferation, accelerated apoptosis, increased G0/G1 phase-arrested cells and decreased S phase-arrested cells, increased ratios of cleaved-caspase3 to pro-caspase3 and of cleaved-caspase9 to pro-caspase9, upregulated protein expression of PARP1, reduced colony and sphere formation, and downregulated expression of Oct4, Nanog and Sox2. However, the cells transfected with ANP32E showed opposite results in these biomarkers. ANP32E upregulation counteracted the inhibitory effect of miR-202-5p mimic in the PCSCs properties (Fig. 3D–J; Additional file 3: Figure S2A–D). In addition, flow cytometry results suggested that overexpression of miR-202-5p or knockdown of ANP32E could significantly reduce the proportion of CD24+ and CD44+ cells. More importantly, further overexpression of ANP32E reversed the inhibiting effect of overexpression of miR-202-5p on the proportion of CD24+ and CD44+ cells (Additional file 4: Figure S3A, B). In summary, miR-202-5p retarded the viability, proliferation, and self-renewal of PCSCs, and accelerated their apoptosis by binding to ANP32E.

### NORAD downregulates miR-202-5p expression in PCSCs

**Fig. 4:**
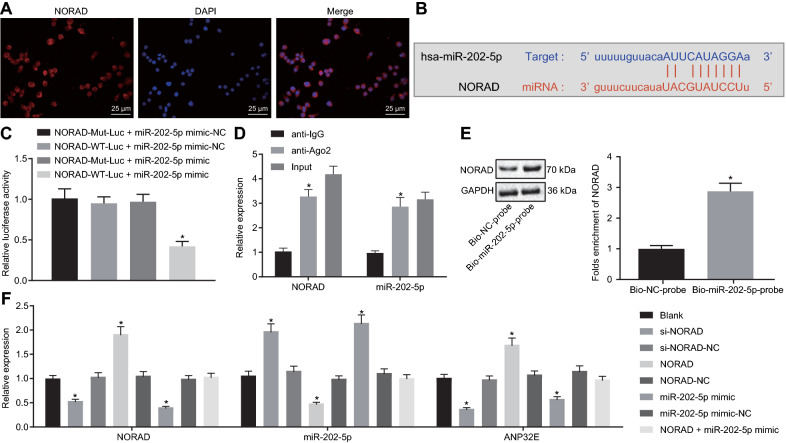
NORAD competitively binds to miR-202-5p and reduces the expression of miR-202-5p in PCSCs. **A** Distribution of NORAD in PC cells (400×); **B** Specific binding sites of NORAD and miR-202-5p; **C** Targeting relationship between miR-202-5p and NORAD measured by dual-luciferase reporter assay (* *p* < 0.05 vs. cells co-transfected with NORAD-WT-Luc and miR-202-5p mimic-NC, NORAD-MUT-Luc and miR-202-5p mimic, NORAD-MUT-Luc and miR-202-5p mimic-NC); **D** NORAD targeted miR-202-5p verified by RIP assay (* *p* < 0.05 vs. IgG pulldown samples); **E** NORAD targeted miR-202-5p verified by RNA pull-down assay (* *p* < 0.05 vs. Bio-NC probe labeled samples); **F** NORAD and miR-202-5p expression as well as the mRNA expression of ANP32E in PCSCs measured by RT-qPCR (* *p* < 0.05 vs. cells without treatment). Measurement data were expressed as mean ± standard derivation. Data between two groups were compared by unpaired *t*-test, and data among multiple groups were analyzed by one-way analysis of variance with Tukey’s post hoc test. The experiment was repeated three times

FISH data illustrated that NORAD was concentrated in the cytoplasm (Fig. 4A). The starBase database predicted miR-202-5p as a downstream gene of NORAD and the presence of binding sites between miR-202-5p and NORAD (Fig. 4B). Meantime, the luciferase activity of the cells co-transfected with NORAD-WT-Luc and miR-202-5p mimic was suppressed (Fig. 4C). The results of RIP showed an enhancement in the expression of NORAD and miR-202-5p in Ago2-pulled samples in comparison with IgG-pulled samples (Fig. 4D). NORAD was found enriched in samples pulled down by the miR-202-5p probe relative to the samples pulled down by the NC probe, as illustrated by RNA pull-down assay data (Fig. 4E).

Transfection efficiency was determined (Additional file 5: Figure S4A). The RT-qPCR results indicated that the expression of NORAD and ANP32E was remarkably decreased in cells after si-NORAD or miR-202-5p mimic treatment, but that of miR-202-5p was increased; the expression of NORAD and ANP32E was augmented in cells transduced with NORAD, while that of miR-202-5p was dramatically reduced. No difference appeared in the expression of NORAD, mIR-202-5p and ANP32E in cells co-transfected with NORAD and miR-202-5p mimic (Fig. 4F). Based on the above results, NORAD competitively inhibited miR-202-5p expression in PCSCs.

### NORAD promotes the viability, proliferation, and stemness of PCSCs through down-regulation of miR-202-5p

**Fig. 5:**
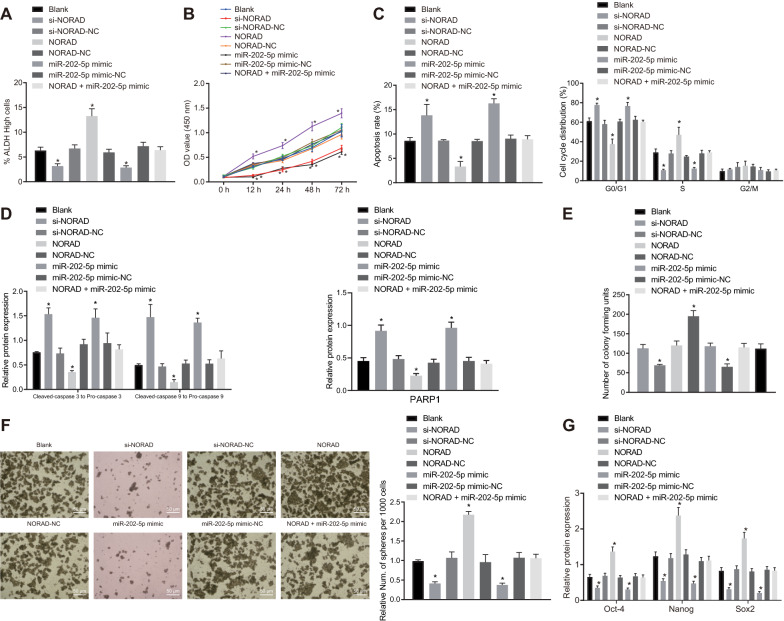
NORAD stimulates the viability, proliferation, and stemness of PCSCs via competitively suppressing miR-202-5p expression. PCSCs were treated with si-NORAD, NORAD and/or miR-202-5p mimic. **A** ALDH activity of PCSCs assessed by Aldefluor assay, where mock means a NC with the addition of DEAB (a specific inhibitor of ALDH enzyme); **B** Proliferation of PCSCs detected by MTT; **C** Apoptosis and cell cycle changes of PCSCs measured by flow cytometry; **D** Protein expression of ratios of cleaved-caspase3 to pro-caspase3, and of cleaved-caspase9 to pro-caspase9, and PARP1 in PCSCs detected by Western blot analysis; **E** Colony formation of PCSCs assessed by colony formation assay; **F** Self-renewal ability of PCSCs detected by sphere formation assay; **G** Protein expression of Oct4, Nanog and Sox2 in PCSCs measured by Western blot analysis; * *p* < 0.05 vs. cells without treatment. Measurement data were expressed as mean ± standard derivation. Data among multiple groups were analyzed by one-way analysis of variance with Tukey’s post hoc test, and data comparison among multiple groups at different time points was conducted using two-way analysis of variance with Tukey’s post hoc test with Bonferroni post hoc test. The experiment was repeated three times

We then aimed to evaluate the role of miR-202-5p and NORAD in PC. Transfection with si-NORAD or miR-202-5p mimic decreased proportion of ALDH high-activity cells, inhibited proliferation, accelerated apoptosis, increased G0/G1 phase-arrested cells and decreased S phase-arrested cells, increased ratios of cleaved-caspase3 to pro-caspase3 and of cleaved-caspase9 to pro-caspase9, upregulated protein expression of PARP1, reduced cell colony and cell sphere formation, and downregulated expression of Oct4, Nanog and Sox2. However, the cells transfected with NORAD exhibited opposite results. Interestingly, the stimulating effects of NORAD on the stemness of PCSCs were partially rescued by miR-202-5p mimic (Fig. 5A–G; Additional file 6: Figure S5A–D). In addition, flow cytometric data indicated that overexpression of miR-202-5p or knockdown of NORAD reduced the proportion of CD24+ and CD44+ cells, while overexpression of NORAD increased the proportion. Overexpression of miR-202-5p abolished the promoting effect of overexpression of NORAD on the proportion of CD24+ and CD44+ cells (Additional file 5: Figure S4B). It is notable that we verified the overexpression efficiency of NORAD by RT-qPCR in MIAPaCa-2 cells (Additional file 7: Figure S6A). As shown in Additional file 7: Figure S6B–H, NORAD overexpression increased the proportion of ALDH high-activity cells, accelerated proliferation, reduced apoptosis, colony, and sphere formation, and G0/G1 phase-arrested cells, increased S phase-arrested cells, and elevated the ratios of cleaved-caspase3 to pro-caspase3 and that of cleaved-caspase9 to pro-caspase9, but downregulated protein expression of PARP1, and upregulated the expression of Oct4, Nanog, and Sox2. To summarize, NORAD induced the viability, proliferation, and self-renewal while suppressing apoptosis of PCSCs through inhibiting miR-202-5p expression.

### NORAD upregulates ANP32E expression to enhance tumorigenicity of PCSCs by competitively inhibiting miR-202-5p in vivo

**Fig. 6:**
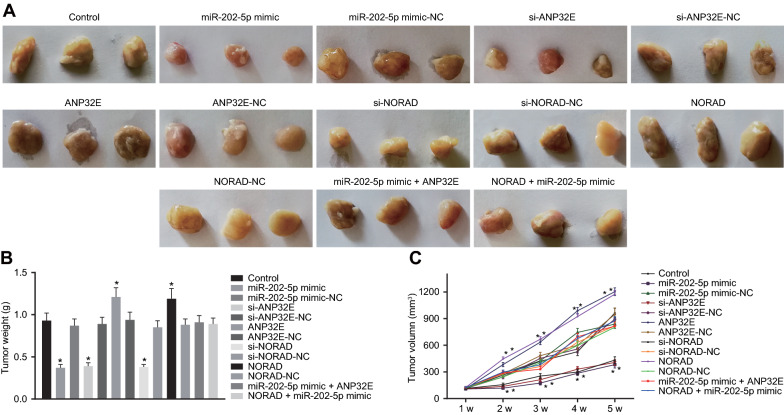
NORAD facilitates the tumorigenicity of PCSCs in vivo by competitively inhibiting miR-202-5p and upregulating ANP32E. **A** Representative images showing xenografts in nude mice treated with miR-202-5p mimic, si-ANP32E, si-NORAD, ANP32E, NORAD, miR-202-5p mimic + ANP32E or NORAD + miR-202-5p mimic. **B** Tumor weight of mice treated with miR-202-5p mimic, si-ANP32E, si-NORAD, ANP32E, NORAD, miR-202-5p mimic + ANP32E or NORAD + miR-202-5p mimic. **C** Tumor volume of mice treated with miR-202-5p mimic, si-ANP32E, si-NORAD, ANP32E, NORAD, miR-202-5p mimic + ANP32E or NORAD + miR-202-5p mimic. * *p* < 0.05 vs. mice without treatment. The above results are all measurements and expressed as mean ± standard derivation. One-way analysis of variance with Tukey’s post hoc test was used for data comparison among multiple groups. Repeated measures analysis of variance was performed for data comparison at different time points with Bonferroni’s post hoc test (n = 12)

To investigate whether and how miR-202-5p overexpression or knockdown of NORAD and ANP32E affected the tumor formation ability, in vivo experiments were applied in nude mice. The tumor volume and weight decreased in the mice treated with miR-202-5p mimic, si-ANP32E and si-NORAD but a contrasting trend appeared following treatments with ANP32E or NORAD. Meanwhile, no alteration occurred in tumor weight and volume of mice injected with PC cells that had been transfected with miR-202-5p mimic-NC, si-ANP32E-NC, ANP32E-NC, NORAD-NC and co-transfected with miR-202-5p mimic and ANP32E or NORAD and miR-202-5p mimic (Fig. 6A–C). The above data conclude that NORAD promoted ANP32E to enhance tumorigenicity of PCSCs via competitive inhibition of miR-202-5p in vivo.

## Discussion

PC denotes a lethal human malignancy around the world in which many patients diagnosed at a late stage [18–20]. Patients, clinicians, and researchers are depressed by the slow progress being made, suggesting that new ideas and solutions to the disease are urgent needed [21–24]. Due to their ability to interact with different structures and molecules, lncRNAs have high heterogeneity and functional diversity [25–27]. As demonstrated previously, lncRNAs function importantly in regulating the cell fate determination, disease occurrence, and tumor progression [28–31]. Thus, this study focused on exploring the regulatory role of NORAD (also known as LINC00657) [32] in the stemness of PCSCs. According to Li et al*.*’s report, NORAD promotes the tumor cell migratory and invasive abilities in pancreatic cancer through modulation on the hsa-miR-125a-3p-metiated RhoA axis [33]. Similarly, as our experiments turned out, NORAD accelerates the viability, proliferation and self-renewal and inhibits apoptosis of PCSCs by impairing expression of miR-202-5p.

Recent investigations uncovered the essential roles of NORAD in biological processes, which also exerts oncogenic functions among various cancers. For instance, as Sun et al*.* discovered, in esophageal squamous cell carcinoma, the acceleration of upregulated NORAD on the cancerous cell invasive, migratory, and proliferative abilities [34]. Wang et al*.* has shed light on the promotive effects of NORAD overexpression on colorectal cancer cell proliferation, migration, and invasion [35]. Based on these researches, aberrantly upregulated NORAD was identified in several human cancers and affect the development of cancers. In our study, the result turned out that NORAD exhibited a significantly high expression in PC cells, and accelerates the cells viability and proliferation, which were in consistency with the research by Li et al*.* [33].

Furthermore, lncRNA could sequester miRNAs to modulate the gene expression [36–39]. Specifically, Tong et al*.* suggested that NORAD downregulation could suppress the cell function of epithelial ovarian cancer by endogenously binding to miR-155-5p [40]. Gao et al*.* indicated the function of NORAD in promoting proliferative ability and glycolysis in non-small cell lung cancer by working as a competing endogenous RNA for miR-136-5p [41]. In addition, it has been found that overexpression of NORAD enhances the invasive and migratory capabilities of melanoma cells via competitive inhibition of miR-205 [42]. Therefore, with the attempt to understand the possible mechanism by which NORAD affecting the PC development, based on the microarray-based analysis, miR-202-5p was screened as a downstream miRNA for NORAD, with specific binding sites identified between them.

Meanwhile, based on the mirDIP, DIANA, TargetScan and starbase databases, ANP32E was revealed as a target gene of miR-202-5p. According to Xiong et al*.* ANP32E has the potency to induce the tumor formation capacity of triple-negative breast cancer cells by transcriptionally potentiating E2F1 [43]. Additionally, knockdown of ANP32E by siRNA lentivirus inhibits the cancerous cell proliferative, migratory, and invasive capabilities in breast cancer [44]. For the purpose evaluating the function of ANP32E in PCSCs, we conducted a wide range of experiments, revealing that NORAD upregulated the ANP32E expression to accelerate the proliferation, self-renewal, and tumorigenic abilities of PCSCs through competitive inhibition of miR-202-5p. In addition, as Chen et al*.* demonstrated in their report, the miR-202 knockout exerts inhibition on the activity of spermatogonial stem cells [45]. Our study suggested that through competitive inhibition of miR-202-5p, NORAD could negatively regulate Oct4, Nanog and Sox2, thus promoting the self-renewal ability of stem cells. Self-renewal is the process of giving rise to indefinitely more cells of the same cell type, perpetuating the stem cell pool throughout life [46]. The stem cells’ self-renew potency is under the regulation of the interaction between intrinsic proteins it expresses and extrinsic signals which it receives from the niche microenvironment [47]. Self-renewal program involves the balance among gate-keeping tumor suppressors (limiting self-renewal), proto-oncogenes (promoting self-renewal), and care-taking tumor suppressors (maintaining genomic integrity) [46].

## Conclusions

**Fig. 7:**
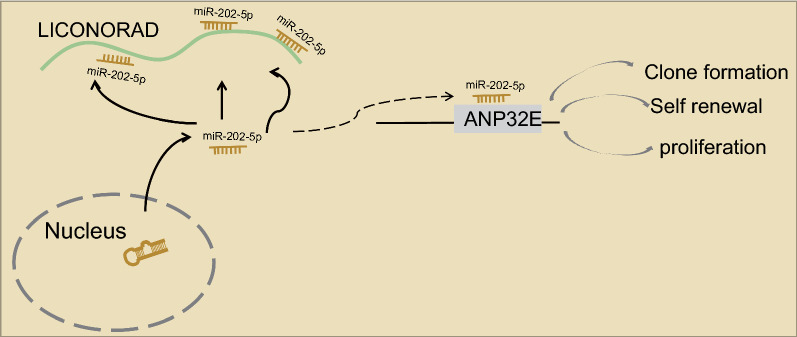
Schematic diagram of the proposed mechanism of the NORAD axis in PCSC tumorgenicity. NORAD upregulates the expression of ANP32E by competitively binding to miR-202-5p, thus accelerating the proliferation and self-renewal of PCSCs

In a word, our investigations offered a new insight into the association between NORAD and PC. In this study, we identified that the promotion of NORAD on the viability, proliferation, and self-renewal of PCSCs, and the inhibition on the apoptosis. Mechanisms suggested that NORAD upregulates ANP32E expression by competitive inhibition of miR-202-5p expression (Fig. 7). Our findings not only highlighted the role of NORAD in PC, but also provided clues for underlying clinical applications. Still, further studies with larger sample size and the presence of metastatic patients are needed to confirm the clinical application of the biomarker of NORAD for the treatment and diagnosis of PC.

## Supplementary Information

**Additional file 1: Table S1.** Primer sequences for RT-qPCR.

**Additional file 2: Figure S1.** Flow cytometric data of ALDH-positive cells in PC cells.

**Additional file 3: Figure S2.** Staining graphs and western blots of Fig. 3. A, ALDH activity of PCSCs assessed by Aldefluor assay, where mock means a NC with the addition of DEAB (a specific inhibitor of ALDH enzyme); B, Apoptosis and cell cycle changes of PCSCs measured by flow cytometry; C, Protein expression of PARP1 and the ratios of cleaved-caspase3 to pro-caspase3 and cleaved-caspase9 to pro-caspase9 in PCSCs detected by Western blot analysis; D, Colony formation of PCSCs assessed by colony formation assay.

**Additional file 4: Figure S3.** miR-202-5p inhibits the self-renewal and stemness of PCSCs by targeting ANP32E. A, Representative images of GFP in PANC-1 cells treated with miR-202-5p mimic, si-ANP32E, ANP32E or miR-202-5p mimic + ANP32E (left) as well as the statistical analysis results (right). B, Flow cytometric analysis of CD24+ and CD44+ cells in PANC-1 cells treated with miR-202-5p mimic, si-ANP32E, ANP32E or miR-202-5p mimic + ANP32E (left) as well as the statistical analysis results (right). * *p* < 0.05 *vs.* cells without treatment.

**Additional file 5: Figure S4.** NORAD promotes the stemness of PCSCs by competitively binding to miR-202-5p. A, Representative images of GFP in cells treated with miR-202-5p mimic, si-NORAD, NORAD or NORAD + miR-202-5p mimic (left) as well as the statistical analysis results (right). B, Flow cytometric analysis of CD24+ and CD44+ cell proportion upon treatment with miR-202-5p mimic, si-NORAD, NORAD or NORAD + miR-202-5p mimic (left) as well as the statistical analysis results (right). * *p* < 0.05 *vs.* cells without treatment.

**Additional file 6: Figure S5.** Staining graphs and western blots of Fig. 5. A, ALDH activity of PCSCs assessed by Aldefluor assay, where mock means a NC with the addition of DEAB (a specific inhibitor of ALDH enzyme); B, Apoptosis and cell cycle changes of PCSCs measured by flow cytometry; C, Protein expression of ratios of cleaved-caspase3 to pro-caspase3, and of cleaved-caspase9 to pro-caspase9, and PARP1 in PCSCs detected by Western blot analysis; D, Colony formation of PCSCs assessed by colony formation assay.

**Additional file 7: Figure S6.** NORAD overexpression facilitates the self-renewal and stemness of PCSCs. A, Overexpression efficiency of NORAD verified by RT-qPCR in MIAPaCa-2 cells. B, ALDH activity of PCSCs assessed by Aldefluor assay, where mock means a NC with the addition of DEAB (a specific inhibitor of ALDH enzyme); C, Proliferation of PCSCs detected by MTT; D, Apoptosis and cell cycle changes of PCSCs measured by flow cytometry; E, Protein expression of PARP1 and the ratios of cleaved-caspase3 to pro-caspase3, and of cleaved-caspase9 to pro-caspase9 in PCSCs detected by Western blot analysis; F, Colony formation of PCSCs assessed by colony formation assay; G, Self-renewal ability of PCSCs detected by sphere formation assay (200 ×); H, Protein expression of Oct4, Nanog, Sox2 in PCSCs measured by Western blot analysis; * *p* < 0.05 *vs.* cells treated with NORAD-NC. Measurement data were expressed as mean ± standard derivation. Data among multiple groups were analyzed by one-way analysis of variance with Tukey's post hoc test, and data comparison among multiple groups at different time points was conducted using two-way analysis of variance with Bonferroni post hoc test. The experiment was repeated three times.

## Data Availability

The datasets generated/analysed during the current study are available.

## Abbreviations

PCSCs: Pancreatic cancer stem cells
CSCs: Cancer stem cells
WT: Wild-type
MUT: Mutant
RIP: RNA binding protein immunoprecipitation
FISH: Fluorescence in situ hybridization
EP: Eppendorf
PC: Pancreatic cancer
lncRNAs: Long non-coding RNAs
miR-202-5p: MicroRNA-202-5p
NORAD: Non-coding RNA activated by DNA damage
ANP32E: Acidic nuclear phosphoprotein 32 family member E
ATCC: American Type Culture Collection
RT-qPCR: Reverse transcription quantitative polymerase chain reaction
si: Small interfering RNA
NCs: Negative controls
DEPC: Diethyl phosphorocyanidate
SSC: Saline sodium citrate
BCIP/NBT: Chromogenic substrate 5-bromo-4-chloro-3ʹ-indoly-phosphate/nitro-blue tetrazolium chloride
GAPDH: Glyceraldehyde-3-phosphate dehydrogenase
ALDH: Aldehyde dehydrogenase
OD: Optical density
PI: Propidium iodide
FITC: Fluorescein isothiocyanate
PE: Phycoerythrin
DMEM: Dulbecco’s modified Eagle’s medium
ANOVA: Analysis of variance
