# Supplementary material for: Long non-coding RNA NORAD promotes pancreatic cancer stem cell proliferation and self-renewal by blocking microRNA-202-5p-mediated ANP32E inhibition
Source: J Transl Med. 2021 Sep 22;19:400. doi: 10.1186/s12967-021-03052-5 (PMC8456629; doi:10.1186/s12967-021-03052-5)
Supplement: Supplementary file 1 — Additional file 1: Table S1. Primer sequences for RT-qPCR. [file 12967_2021_3052_MOESM1_ESM.docx]

**Supplementary Table 1** Primer sequences for RT-qPCR

| Target | Sequence (5'-3') |
| --- | --- |
| NORAD | F: 5'-TGATAGGATACATCTTGGACATGGA-3' |
|  | R: 5'-AACCTAATGAACAAGTCCTGACATACA-3' |
| miR-202-5p | F: 5'-TTCCTATGCATATACTTCTTTG-3' |
|  | R: universal primer |
| ANP32E | F: 5'-TGCCTGTGTGTCAATGGGG-3' |
|  | R: 5'-GCAGAGCTTCTACTGTACTGAGA-3' |
| U6 | F: 5'-CTCGCTTCGGCAGCACA-3' |
|  | R: 5'-AACGCTTCACGAATTTGCGT-3' |
| GAPDH | F: 5'-TAACTCTGGTAAAGTGGATATTG-3' |
|  | R: 5'-GAAGATGGTGATGGGATTTC-3' |

Note: RT-qPCR, transcription quantitative polymerase chain reaction; NORAD, non-coding RNA activated by DNA damage; ANP32E, acidic nuclear phosphoprotein 32 family member E; PARP1, poly ADP-ribose polymerase; Oct4, octamer-binding transcription factor-4; Sox2, SRY-box containing gene 2; GAPDH, glyceraldehyde-3-phosphate dehydrogenase.
